# Prolonged contextual fear memory in AMPA receptor palmitoylation-deficient mice

**DOI:** 10.1038/s41386-022-01347-9

**Published:** 2022-05-26

**Authors:** Akiko Oota-Ishigaki, Keizo Takao, Daisuke Yamada, Masayuki Sekiguchi, Masayuki Itoh, Yumie Koshidata, Manabu Abe, Rie Natsume, Masaki Kaneko, Toma Adachi, Toshie Kaizuka, Nami Suzuki, Kenji Sakimura, Hiroyuki Okuno, Keiji Wada, Masayoshi Mishina, Tsuyoshi Miyakawa, Takashi Hayashi

**Affiliations:** 1 Biomedical Research Institute, National Institute of Advanced Industrial Science and Technology (AIST), Tsukuba, Ibaraki 305-8566 Japan; 2 Section of Behavior Patterns, Center for Genetic Analysis of Behavior, National Institute for Physical Sciences (NIPS), Okazaki, Aichi 444-8585 Japan; 3 Life Science Research Center, University of Toyama, Toyama, 930-0194 Japan; 4 National Institute of Neuroscience, National Center of Neurology and Psychiatry (NCNP), Kodaira, Tokyo, 187-8502 Japan; 5 Department of Cellular Neurobiology, Brain Research Institute, Niigata University, Niigata, 951-8585 Japan; 6 Medical Innovation Center/SK Project, Graduate School of Medicine, Kyoto University, Kyoto, 606-8507 Japan; 7 Department of Biochemistry and Molecular Biology, Kagoshima University Graduate School of Medical and Dental Sciences, Kagoshima, 890-8544 Japan; 8 Brain Science Laboratory, The Research Organization of Science and Technology, Ritsumeikan University, Kusatsu, Shiga 525-8577 Japan; 9 Division of Systems Medical Science, Institute for Comprehensive Medical Science, Fujita Health University, Toyoake, Aichi 470-1192 Japan

**Keywords:** Post-traumatic stress disorder, Trauma

## Abstract

Long-lasting fear-related disorders depend on the excessive retention of traumatic fear memory. We previously showed that the palmitoylation-dependent removal of synaptic α-amino-3-hydroxy-5-methyl-4-isoxazole propionate (AMPA) receptors prevents hyperexcitation-based epileptic seizures and that AMPA receptor palmitoylation maintains neural network stability. In this study, AMPA receptor subunit GluA1 C-terminal palmitoylation-deficient (GluA1C811S) mice were subjected to comprehensive behavioral battery tests to further examine whether the mutation causes other neuropsychiatric disease-like symptoms. The behavioral analyses revealed that palmitoylation-deficiency in GluA1 is responsible for characteristic prolonged contextual fear memory formation, whereas GluA1C811S mice showed no impairment of anxiety-like behaviors at the basal state. In addition, fear generalization gradually increased in these mutant mice without affecting their cued fear. Furthermore, fear extinction training by repeated exposure of mice to conditioned stimuli had little effect on GluA1C811S mice, which is in line with augmentation of synaptic transmission in pyramidal neurons in the basolateral amygdala. In contrast, locomotion, sociability, depression-related behaviors, and spatial learning and memory were unaffected by the GluA1 non-palmitoylation mutation. These results indicate that impairment of AMPA receptor palmitoylation specifically causes posttraumatic stress disorder (PTSD)-like symptoms.

## Introduction

While fear memory is effective in avoiding dangerous situations, which promotes survival in complex and dynamically changing environments, fear extinction processes need to occur in parallel to suppress excessive fear and anxiety [1–3]. Posttraumatic stress disorder (PTSD) is a mental and behavioral disorder that is triggered by either experiencing or witnessing a traumatic event. Recollection of terrifying fear usually occurs when patients are exposed to incentives. Failure of appropriate fear reduction leads to PTSD and afflicts a person throughout their life span. Uncontrollable augmentation of synaptic transmission in neuronal fear pathways has been hypothesized to be involved in anxiety-related disorders including PTSD and impair the regulation of excitatory synapses in fear-related amygdala regions; their interactions with the hippocampus and prefrontal cortex (PFC) may play a role in the pathophysiology of PTSD [4, 5].

Glutamate is the major excitatory neurotransmitter in the mammalian central nervous system. The expression of postsynaptic α-amino-3-hydroxy-5-methyl-4-isoxazole propionate (AMPA)-type ionotropic glutamate receptors (AMPA receptors) is closely linked to excitatory synaptic strength [6, 7]. Therefore, the quantitative control of synaptic AMPA receptor numbers is critical for basal synaptic transmission, synaptic plasticity, and higher brain function [7–10]. Among the four AMPA receptor subunits (GluA1, 2, 3, and 4, also known as GluR1-4, GluRA-D, or GluRα1–4), GluA1 has a dominant role during activity-dependent AMPA receptor insertion into synapses [11]. AMPA receptor trafficking to and from synapses is dynamically regulated by post-translational protein modifications such as phosphorylation [11–13]. In these processes, AMPA receptor phosphorylation reversibly modulates the properties of AMPA receptor ion channels and membrane trafficking of AMPA receptors to the postsynaptic membrane [7, 14–16]. Previous studies have shown that phosphorylation of AMPA receptors regulates various forms of fear memory [17, 18].

Another key modification of AMPA receptors is reversible *S*-palmitoylation, the covalent attachment of palmitic acid to intracellular cysteine residues via thioester bonds [19–22]. Generally, palmitoylation acts as a sticky tag that can direct proteins, including many neuronal receptors and ion channels, to specific regions on the plasma membrane or specific intracellular membranes or vesicles [23–25]. We have previously reported that palmitoylation regulates the synaptic expression of AMPA receptors [16, 26–30]. All mammalian AMPA receptor subunits, GluA1-4, are palmitoylated at their C-terminal conserved region in an activity-dependent manner [24, 26, 31, 32]. Palmitoylation inhibits GluA1 interaction with the postsynaptic 4.1N protein, which stabilizes synaptic AMPA receptor expression in long-term potentiation (LTP) [26, 27, 33]. We recently generated mice lacking the palmitoylation site of GluA1 at Cys811 by substituting with Ser (GluA1C811S) and demonstrated that a deficiency in GluA1 palmitoylation enhanced seizure susceptibility and robust LTP-induced spine enlargement without affecting gross brain structure and normal excitatory synaptic transmission [34, 35]. Furthermore, the mutation at the GluA1 palmitoylation site induces hyperexcitation-based epileptic seizures, and the anticonvulsive effects of clinically used antiepileptic drugs were reduced, which suppressed excess excitation [36]. Our findings indicate that an abnormality in palmitoylation-dependent regulation of the AMPA receptor may lead to hyperexcitability, which weakens the maintenance of network stability throughout the brain. In summary, palmitoylation appears uniquely suited to create dynamic quantitative control of synaptic receptor numbers and intracellular trafficking of AMPA receptors, which are associated with complex neuronal events [37].

Here, we further analyzed GluA1C811S knock-in mice on a pure C57BL/6N genetic background to examine whether the palmitoylation-deficient mutation causes other neuropsychiatric disease-like symptoms. The effects of palmitoylation site ablation on behavior were examined using a comprehensive behavioral test battery. The results revealed several characteristic features of GluA1C811S mice, including prolonged fear memory, whereas they showed no significant alteration of anxiety-like behaviors at the basal state.

## Materials and methods

### Animals and design of behavioral experiments

GluA1C811S mutant mice were backcrossed into the C57BL/6N strain (Charles River Laboratories Japan, Inc.) at least five times. The GluA1C811S allele was identified by PCR, as previously described [34]. The intercross of heterozygotes resulted in the production of wild-type (wt), heterozygous, and homozygous offspring at the expected 1:2:1 Mendelian ratio. Only male mice were used for the subsequent behavioral analyses.

We prepared two independent groups of mice for behavioral battery testing. All behavioral tests were carried out with male mice that were 10–12 (first group) or 28–34 (second group) weeks old at the start of testing. Dubious differences observed in the first group were double-checked using the second group. Different age groups were used to confirm that these behavioral changes were induced by GluA1 palmitoylation deficiency regardless of age. Male mice were housed in groups of four (two pairs of wt and GluA1C811S knock-in mice) per standard animal cage in a room under a 12-h light/dark cycle with access to standard laboratory chow and water *ad libitum*. All experimental procedures, except measurements of body weight and body temperature, were performed in a soundproof room. Prior to all experiments, the mice were left undisturbed in the testing room for at least 30 min to allow for acclimation. The order of the tests is listed in Table 1. Our serial behavioral tests have been designed from least to most invasive and from less to most burdened with recovery time between tests to decrease the chance that behavioral responses are influenced by prior test history [38–43]. Each behavioral test was separated from the next one by at least 1 day. After each test, the entire apparatus was cleaned with a diluted sodium hypochlorite solution to prevent bias due to olfactory cues. All behavioral tests were conducted as previously described [44–47]. Even a little suspicious phenotypes observed in first group were reconfirmed by testing in second group.

**Table 1:** Comprehensive behavioral test battery for GluA1C811S mutant mice.

1st group (wild-type, *n* = 20; GluA1C811S, *n* = 20)				2nd group (wild-type, *n* = 17; GluA1C811S, *n* = 19)			
Test	Age (weeks old)	Days	Results	Test	Age (weeks old)	Days	Results
GHNS	10–12	1–2		GHNS	28–34	1–2	Table 2
LD	10–12	3		LD	28–34	3	Fig. 1A
OF	11–13	7	Fig. 1B Supplementary Fig. 2A	HP	28–34	4	Table 2
EP	11–13	8	Fig. 1C	RR	28–35	8	Supplementary Fig. 1A
HP	11–13	9		PPI	29–36	14	Table 2
SI	11–13	10	Supplementary Fig. 3A	PS	29–36	15–16	Supplementary Fig. 4A
RR	12–14	15					
CSI	13–15	21	Supplementary Fig. 3B				
PS	13–15	24					
GA	14–16	30	Supplementary Fig. 1C				
HP (2nd)	14–16	31					
BM	16–23	46–88	Supplementary Fig. 5A				
NIH	23–25	102	Fig. 1D				
BT	24–26	110	Supplementary Fig. 1B				
TM-SA	25–27	117	Supplementary Fig. 5B				
PaS	26–28	122	Supplementary Fig. 5C				
TS	26–28	123	Supplementary Fig. 4B				
Obj Res	27–29	128					
Obj Rec	28–30	133					
PPI	28–30	134–135					
FZ	28–35	136–170	Fig. 2AB				
FE	35–51	186–275	Fig. 2CD Supplementary Fig. 6				
OF (2nd)	38–40	196					
HCSI	38–41	199–207	Supplementary Fig. 3C				

The order of tests was as follows: first group (wild-type, *n* = 20; GluA1C811S, *n* = 20): general health and neurological screen (GH), neuromuscular strength examination (NS), light/dark transition test (LD), open field test (OF), elevated plus maze test (EP), hot plate test (HP), social interaction test in a novel environment (SI), rotarod test (RR), Crawley’s sociability and preference for social novelty (three-chamber) test (CSI), Porsolt forced swim test (PS), gait analysis (GA), 2nd hot plate test (HP), Barnes maze test (BM), Novelty-induced hypophagia test (NIH), beam test (BT), T-maze spontaneous alteration test (TM-SA), pattern separation test (PaS), tail suspension test (TS), object reaction response test (Obj Res), object recognition and object recency test (Obj Rec), startle response/prepulse inhibition test (PPI), contextual and cued fear conditioning test (FZ), fear erase test (FE), 2nd open field test (OF), and social interaction in home cage (HCSI); second group (wild-type, *n* = 17; GluA1C811S, *n* = 19): general health and neurological screen (GH), neuromuscular strength examination (NS), light/dark transition test (LD), hot plate test (HP), rotarod test (RR), startle response/prepulse inhibition test (PPI), and Porsolt forced swim test (PS).

All animal care procedures and experiments were performed in accordance with the regulations and institutional guidelines of the National Center of Neurology and Psychiatry (NCNP), National Institute for Physiological Sciences (NIPS), and National Institute of Advanced Industrial Science and Technology (AIST). The technical protocols for animal experiments in this study were approved by the Animal Care and Use Committees of NCNP, NIPS, and AIST. Raw data from the behavioral tests, the date on which each experiment was performed, and the age of each mouse at the time of the experiment are available from the Mouse Phenotype Database (http://www.mouse-phenotype.org/). The detailed protocols of the comprehensive behavioral battery tests are provided in the Supplementary Information.

### Electrophysiology

Preparation of basolateral amygdala (BLA) slices and whole-cell recordings were performed as described previously with minor modifications [34, 35]. AMPA/NMDA ratios were calculated as the ratio of the peak AMPAR-current at −70 mV to the NMDAR-current 80 ms after stimulus onset at +40 mV. The detailed protocols are provided in the Supplementary Information.

### Biochemical analysis

Palmitoylation of GluA1 protein was assessed using the acyl-biotinyl exchange (ABE) method as described previously [34]. The detailed protocols are provided in the Supplementary Information.

### Statistical analysis

The analysis was conducted using StatView (SAS Institute, Cary, NC, USA) or SPSS (IBM, Chicago, IL, USA). Data were analyzed by one-way ANOVA followed by Tukey’s test, two-way repeated measures ANOVA followed by Fisher’s LSD test, ANCOVA, MANOVA, Student’s *t*-tests, paired *t*-tests, Mann-Whitney’s *U* test, or log-rank test. Statistical significance was set at *p* < 0.05.

## Results

### Normal appearance of GluA1C811S mutant mice

We subjected homozygotes of GluA1C811S mice and their wt littermates to a comprehensive battery of behavioral tests to evaluate the behavioral effects of deficiency of C-terminal palmitoylation of GluA1 (Table 1) [48]. As we previously reported [34], GluA1C811S mice appeared healthy and showed no obvious differences in their physical characteristics (Table 2). There were no significant differences between the genotypes in body weight, neuromuscular strength, startle response, prepulse inhibition, or pain sensitivity (Table 2). Non-palmitoylation C811S mutation in GluA1 did not seriously affect locomotor ability (Supplementary Fig. 1A, B), with mild changes in bowlegged-walking habits in GluA1C811S mice (Supplementary Fig. 1C). In accordance with our previous observation [34], there were also no significant differences between wt and GluA1C811S mice at the basal level without intense shock. Furthermore, GluA1C811S mice showed normal sociability (Supplementary Fig. 3, see details below), normal spatial memory, and normal pattern separation (Supplementary Fig. 5, see details below).

**Table 2:** General physical characteristics and sensory and motor functions of wild-type and GluA1C811S mutant mice.

Test				wild-type	GluA1C811S	*p* value
Physical characterization	body weight (g)			43.6 ± 1.1	43.4 ± 0.9	0.91
	rectal temperature (°C)			34.3 ± 0.2	34.1 ± 0.2	0.46
Neuromuscular strength	grip strength (N)			0.68 ± 0.03	0.63 ± 0.03	0.26
	wire hang (s)			4.84 ± 0.54	4.02 ± 1.04	0.50
Sensory function	acoustic startle response (a.u.)		110 dB	0.76 ± 0.10	0.95 ± 0.13	0.50
			120 dB	1.01 ± 0.12	1.05 ± 0.13	
	prepulse inhibition (%)		74–110 dB	41.0 ± 8.0	42.7 ± 6.6	0.83
			78–110 dB	60.0 ± 5.3	61.5 ± 3.8	
			74–120 dB	31.4 ± 6.2	26.5 ± 7.9	0.51
			78–120 dB	55.2 ± 4.8	49.9 ± 5.4	
Hot plate	latency to avoid (s)			7.24 ± 0.58	7.94 ± 0.64	0.43

The *p* values represent the genotype effect in the ANOVA. All values are represented as the mean ± SEM.

### Unaffected anxiety-like behaviors in GluA1C811S mutant mice

**Fig. 1:**
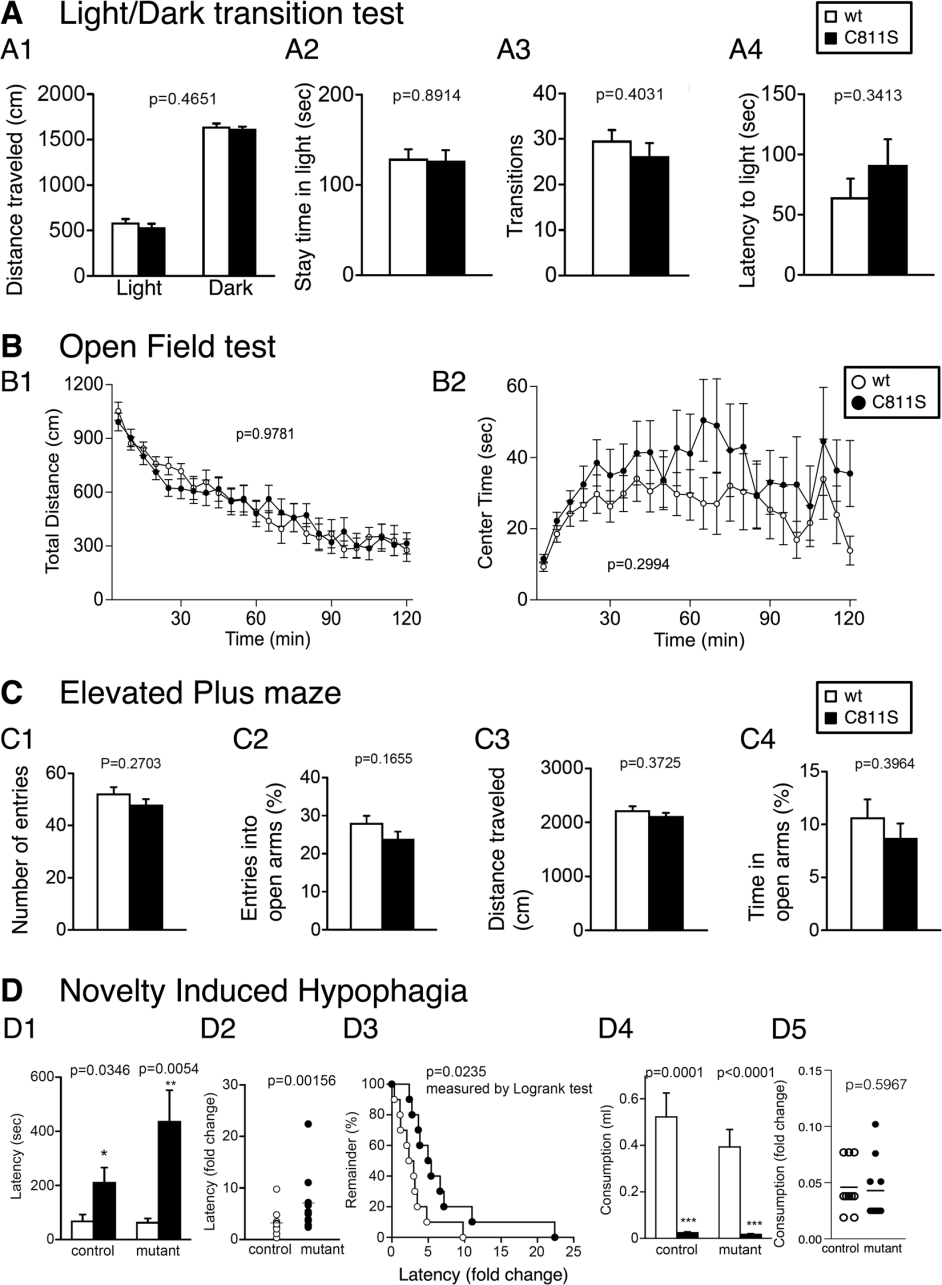
Normal anxiety-like behaviors in GluA1C811S mutant mice. **A** Light/dark transition test: distance traveled in the light and dark box (A1), time spent in the light chamber (s) (A2), number of transitions between the light and dark box (A3), and latency time before the first entry into the light box (s) (A4). **B** Open field test: total locomotion distance traveled (cm) (B1) and time spent in the center (s) (B2). **C** Elevated plus maze: number of entries into the center crossing between the open and closed arms (C1), percentage of entries into the open arms (C2), total distance traveled (cm) (C3), and percentage of time spent on the open arms (C4). **D** Novelty-induced hypophagia test: latency to begin drinking in the home (white) and novel (black) cage (D1), difference in latency between novel and home cage environments (D2, D3), consumption in the home (white) and novel (black) cage (D4), and difference in consumption between novel and home cage environments (D5). All data are expressed as mean ± SEM. The *p* values indicate genotype effects.

We then performed a series of tests on anxiety-like behaviors at the basal level [49, 50]. In the light/dark transition test, there were no significant differences between the genotypes in distance traveled (Fig. 1A1; *F*_1, 34_ = 0.546, *p* = 0.4651), time spent in the light chamber (Fig. 1A2; *F*_1, 34_ = 0.019, *p* = 0.8914), number of transitions between chambers (Fig. 1A3; *F*_1, 34_ = 0.717, *p* = 0.4031), and first latency to enter the light chamber (Fig. 1A4; *F*_1, 34_ = 0.931, *p* = 0.3413). Spontaneous locomotor activity was examined using an open field test (Fig. 1B). No obvious differences were observed between genotypes in horizontal activity (Fig. 1B1; *F*_1, 38_ = 0.001, *p* = 0.9781), time spent in the center area (Fig. 1B2; *F*_1, 38_ = 1.107, *p* = 0.2994), vertical activity (Supplementary Fig. 2A1; *F*_1, 38_ = 0.169, *p* = 0.683), and stereotypic behaviors (Supplementary Fig. 2A2; *F*_1, 38_ = 0.137, *p* = 0.713). In the elevated plus maze test, the GluA1C811S mice’s behavior was similar to that of their wt littermates in number of entries into the arms (Fig. 1C1; *F*_1, 38_ = 1.251, *p* = 0.2703), percentage of entries into the open arms (Fig. 1C2; *F*_1, 38_ = 2.000, *p* = 0.1655), distance traveled (Fig. 1C3; *F*_1, 38_ = 0.814, *p* = 0.3725), and percentage of time spent in the open arms (Fig. 1C4; *F*_1, 38_ = 0.736, *p* = 0.3964). In the novelty-induced hypophagia test, the latency to begin drinking water in a novel cage was greater than that in the home cage in both genotypes (Fig. 1D1; control: *p* = 0.0346, mutant: *p* = 0.0054), and the fold change in consumption was larger in GluA1C811S mice than in wt mice (Fig. 1D2; *p* = 0.00156 and Fig. 1D3; *p* = 0.0235, log-rank test). Consumption in the novel cage was lower than that in the home cage in both genotypes (Fig. 1D4; control: *p* = 0.0001, mutant: *p* < 0.0001), but no significant difference was observed in the fold change of consumption (Fig. 1D5; *p* = 0.5967). These results suggest that anxiety-like behavior is not affected by Cys to Ser non-palmitoylation mutation in GluA1 in daily activities.

### Normal social behaviors in GluA1C811S mutant mice

In the social interaction test conducted in a novel environment, the total duration of contacts (Supplementary Fig. 3A1; *F*_1, 18_ = 0.246, *p* = 0.6261), number of contacts (Supplementary Fig. 3A2; *F*_1, 18_ = 0.002, *p* = 0.962), total duration of active contacts (Supplementary Fig. 3A3; *F*_1, 18_ = 0.005, *p* = 0.9417), mean duration per contact (Supplementary Fig. 3A4; *F*_1, 18_ = 0.991, *p* = 0.3327), and distance traveled (Supplementary Fig. 3A5; *F*_1, 18_ = 0.112, *p* = 0.7415) did not differ between genotypes. In Crawley’s sociability and preference for social novelty test (three-chamber test), we did not find any differences between the genotypes in the sociability indices (Supplementary Fig. 3B1 left: ratio of stay time; *F*_1, 38_ = 0.045, *p* = 0.8339, right: distance traveled; *F*_1, 38_ = 0.872, *p* = 0.3563), and the social novelty preference test (Supplementary Fig. 3B2 left: ratio of stay time; *F*_1, 38_ = 0.026, *p* = 0.8718, right: distance traveled; *F*_1, 38_ = 0.021, *p* = 0.886). We also monitored social interactions in the home cage under familiar conditions over a 7-day period. In the social interaction test in the home cage, time spent separated usually increases when mice are active and decreases when mice are sleeping. There were no significant differences between the genotypes in the mean number of detected particles (Supplementary Fig. 3C1 top; night period: *F*_1, 15_ = 0.565, *p* = 0.464, day period: *F*_1, 15_ = 0.02, *p* = 0.8897, total: *F*_1, 15_ = 0.234, *p* = 0.6359), indicating that GluA1C811S mice displayed normal social interaction behavior in their home cages. Locomotor activity in the home cage did also not differ between the genotypes (Supplementary Fig. 3C1 bottom; night period: *F*_1, 15_ = 0.009, *p* = 0.9274; day period: *F*_1, 15_ = 0.033, *p* = 0.8573, total: *F*_1, 15_ = 0.017, *p* = 0.8967). The average 3-day moving pattern (days 3–5), is depicted in Supplementary Fig. 3C2. There were no significant differences between the genotypes in the mean number of particles (Supplementary Fig. 3C2 top; night period: *F*_1, 15_ = 1.181, *p* = 0.2943, day period: *F*_1, 15_ = 0.154, *p* = 0.7003, total: *F*_1, 15_ = 0.189, *p* = 0.6698) or locomotor activity in the home cage (Supplementary Fig. 3C2 bottom; night period: *F*_1, 15_ = 0.11, *p* = 0. 7444; day period: *F*_1, 15_ = 0.308, *p* = 0.5869, total: *F*_1, 15_ = 0.004, *p* = 0.951).

### Unaffected depression-related behaviors in GluA1C811S mutant mice

Two types of experiments related to depression-related behaviors were conducted. GluA1C811S mice showed immobility similar to wt mice in inescapable stressful environments (Supplementary Fig. 4). In the Porsolt forced swim test, there were no significant differences between the genotypes in immobility (Supplementary Fig. 4A, top: *F*_1, 34_ = 0.121, *p* = 0.7299 on the first day; *F*_1, 34_ = 1.791, *p* = 0.1896 on the second day) or distance traveled (Supplementary Fig. 4A, bottom; *F*_1, 34_ = 0.927, *p* = 0.3424 on the first day; *F*_1, 34_ = 0.731, *p* = 0.3984 on the second day). In the tail suspension test, GluA1C811S mice showed immobility results that were similar to those of their wt littermates (Supplementary Fig. 4B; *F*_1, 37_ = 0.788, *p* = 0.3805).

### Normal spatial learning and memory in GluA1C811S mutant mice

Concerning learning and memory, we first examined spatial reference memory using the Barnes maze test and spatial working memory using the T-maze test (Supplementary Fig. 5A, B). In the Barnes circular maze, there was no significant effect of genotype on the number of search errors made during acquisition (Supplementary Fig. 5A1 left; *F*_1, 38_ = 2.004, *p* = 0.1651) or the latency to find the target hole (Supplementary Fig. 5A1 right; *F*_1, 38_ = 0.13, *p* = 0.7208), indicating normal acquisition of spatial reference memory in GluA1C811S mice. Probe trials in which the escape box was removed were performed 1 day (1st test) and 30 days (2nd test) after the last day of training. During the probe trial, both genotypes showed a significant effect of hole location both in the 1st and 2nd tests, indicating that both genotypes recalled the location of the target. There were no significant differences between the genotypes in the time spent around the target during the 1st (Supplementary Fig. 5A2; *F*_1, 38_ = 4.27, *p* = 0.0457) and 2nd tests (Supplementary Fig. 5A3; *F*_1, 38_ = 2.738, *p* = 0.1062). The results of the probe trials suggest that GluA1C811S mice have intact consolidation or retention of spatial reference memory.

We then examined behavioral flexibility using reversal tasks. The mice were trained for an additional 4 days after the 2nd probe test. The target was then moved to the opposite site. During the reversal training, there was no significant difference in the number of errors (Supplementary Fig. 5A4 left; *F*_1, 38_ = 0.339, *p* = 0.5636), whereas the latency to find the target hole was slightly larger in GluA1C811S mice than in wt mice (Supplementary Fig. 5A4 right; *F*_1, 38_ = 1.909, *p* = 0.1752). In the probe test after the reversal training, both wt and GluA1C811S mice spent a similar time around the target hole (Supplementary Fig. 5A5; *p* = 0.1468, one-way ANOVA). Thus, GluA1C811S mice exhibited comparable behavioral flexibility to wt mice.

In the T-maze spontaneous alternation task, both wt and GluA1C811S mice showed a similar performance (Supplementary Fig. 5B; *F*_1, 38_ = 0.416, *p* = 0.5229).

Pattern separation ability was also examined using the non-associative place-learning test. Both genotypes showed significantly reduced motility in the combination of the pattern (Supplementary Fig. 5C; *F*_3, 36_ = 10.458, *p* < 0.0001 for wt, *F*_3, 36_ = 6.439, *p* = 0.0005 for GluA1C811S), while there was no significant reduction in the different-combination groups (Supplementary Fig. 5C; *p* = 0.1116 for wt, *p* = 0.1353 for GluA1C811S). The results suggest that both genotypes can similarly distinguish pattern differences.

### Enhanced acquisition of contextual, but not cued, fear memory long-lasting in GluA1C811S mutant mice

**Fig. 2:**
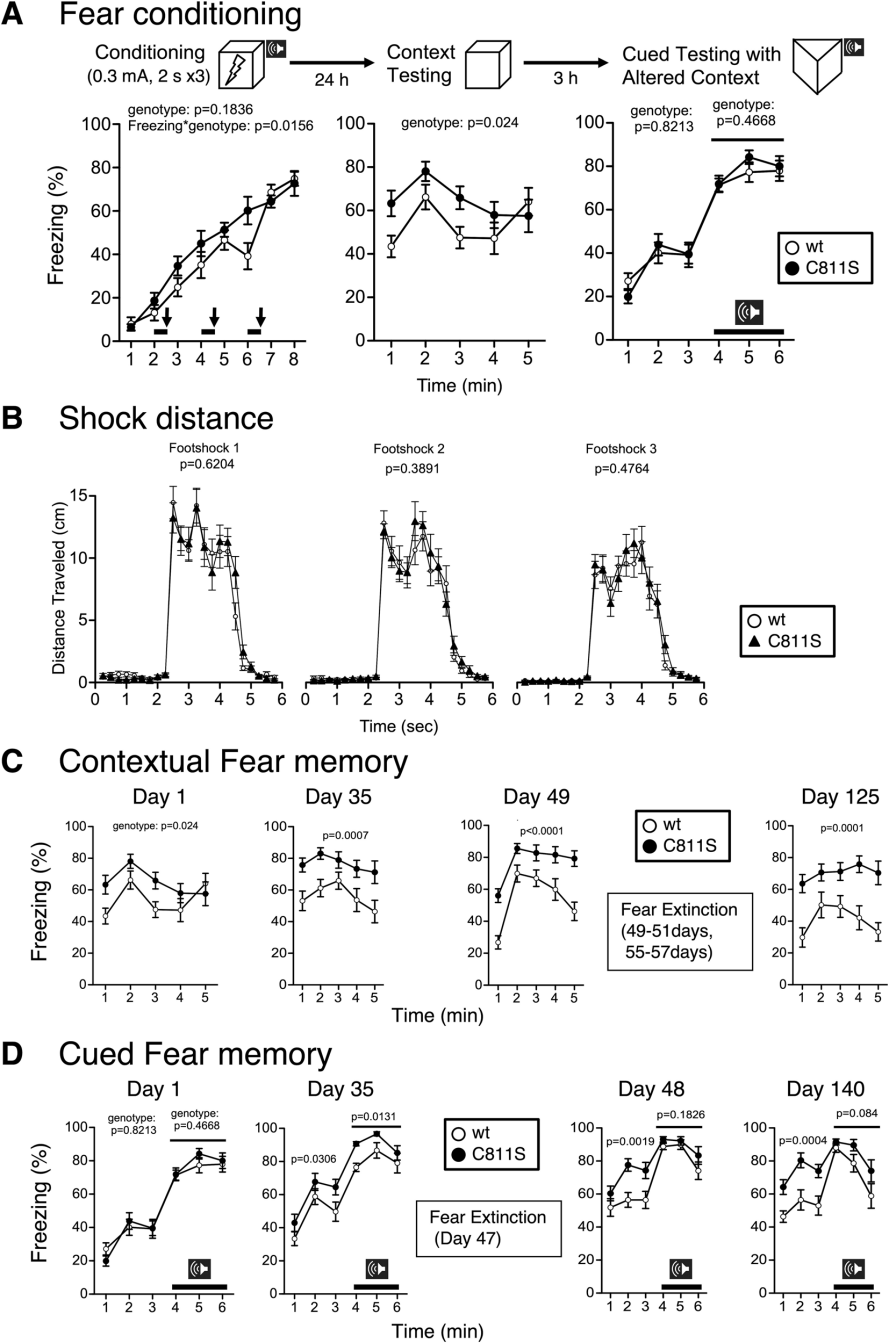
Enhanced acquisition of contextual, but not cued, fear memory in GluA1C811S mutant mice. **A** Freezing ratios in fear conditioning (left) and contextual test (middle) or cued test with altered context (right) 1 day after conditioning. **B** Shock distances after each shock. **C** Contextual fear memory at 1, 35, 49, or 125 days after conditioning. **D** Cured fear memory at 1, 35, 48, or 140 days after conditioning. Bold lines and arrows represent tone and footshock, respectively. All data are expressed as mean ± SEM. The *p* values indicate genotype effects.

Finally, we examined the fear memory of GluA1C811S mice using contextual and cued fear conditioning tests. During the conditioning period, freezing behavior before the first presentation of cue-shock pairings was minimal and did not differ between wt and GluA1C811S mice. After footshocks, freezing responses of both genotypes were similarly increased (genotype effect, *F*_1, 38_ = 1.835, *p* = 0.1836; genotype × time effect, *F*_7, 266_ = 2.528, *p* = 0.0156) (Fig. 2A, left). Correspondingly, both genotypes showed similar moving patterns after each shock (Fig. 2B) and comparable pain sensitivity (Table 2, hot plate test). One day after conditioning, the freezing level of GluA1C811S mice was significantly higher than that exhibited by wt mice in the contextual test (Fig. 2A, middle; genotype effect, *F*_1, 38_ = 5.532, *p* = 0.024). In contrast, there were no differences between genotypes in cue (tone)-dependent (auditory) fear conditioning (Fig. 2A, right; genotype effect, *F*_1, 38_ = 0.052, *p* = 0.8213, 1–3 min; genotype effect, *F*_1, 38_ = 0.54, *p* = 0.4668, 4–6 min).

Thirty-five or 49 days after conditioning, contextual fear memory scores were still significantly higher in GluA1C811S mice than in wt mice (genotype effect, *F*_1, 38_ = 13.644, *p* = 0.0007 at 35 days, *F*_1, 38_ = 30.952, *p* < 0.0001 at 49 days). The contextual fear enhancement in GluA1C811S mice was still observed 4 months later, even after standard fear extinction re-exposure training (genotype effect, *F*_1, 38_ = 18.872, *p* = 0.0001 at 125 days) (Fig. 2C). Concerning cued fear memory, GluA1C811S mice showed an enhancement of freezing responses at 35 days, just induced by transferring them from the home cage, even though fear acquisition was not influenced by GluA1 C-terminal palmitoylation. Interestingly, this generalized fear response sustained for 140 days, even after fear extinction training (Fig. 2D and Supplementary Fig. 6). These results demonstrate the strong formation of fear generalization in GluA1C811S mice [51, 52].

### Impaired extinction and elevated excitation in the BLA of GluA1C811S mutant mice

**Fig. 3:**
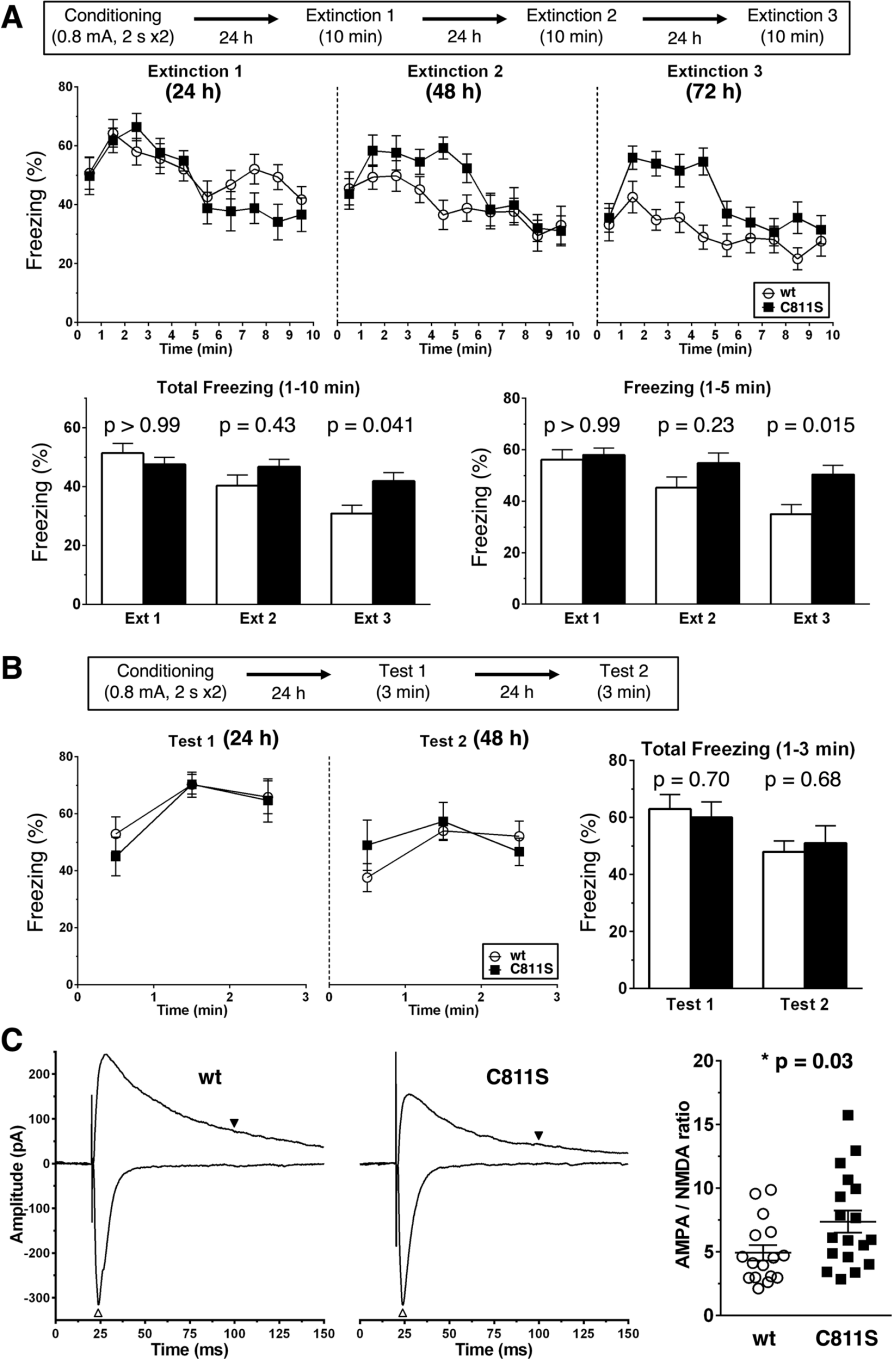
Impaired extinction of fear memory in GluA1C811S mutant mice. **A** Contextual fear memory was erased by repeated exposure for 10 min to the conditioned stimulus at 24, 48, or 72 h after fear conditioning (wt: *n* = 17 mice, C811S: *n* = 13 mice). **B** Contextual fear memory was erased by repeated exposure for 10 min to the conditioned stimulus at 24 or 48 h after fear conditioning (wt: *n* = 8 mice, C811S: *n* = 5 mice). **C** AMPA/NMDA ratio. Basolateral amygdala (BLA) pyramidal neurons were held at either −70 mV or +40 mV, and EPSCs evoked by the stimulation were recorded (left). White and black arrowheads indicate AMPA receptor- and NMDA receptor-mediated currents used for calculation of AMPA/NMDA ratio, respectively. AMPA/NMDA ratios were shown (right) (wt: *n* = 16 cells from 4 mice, C811S: *n* = 18 cells from four mice). All data are expressed as mean ± SEM. Two-way ANOVA was conducted, followed by Bonferroni test (**A**, **B**). The *p* values indicate genotype effects.

After a comprehensive battery of behavioral tests, we further investigated the basis of the poor extinction of contextual fear memory in GluA1C811S mice [53–55]. Repeated exposure of wt mice to the conditioned chamber for 10 min at 24, 48, and 72 h after the footshock gradually decreased freezing rates, but these extinction procedures had less effect on GluA1C811S mice (Fig. 3A). The differences were remarkable during the first 5 min. Shorter exposure to the conditioned chamber for 3 min had little effect on either genotype (Fig. 3B).

As for the contextual fear memory-related neural circuit, the C811S non-palmitoylation mutation in GluA1 led to increase the ratio of AMPA receptor- to NMDA receptor-mediated synaptic currents in BLA pyramidal neurons (Fig. 3C). We then biochemically confirmed the mutation of the palmitoylation site by an acyl-biotinyl exchange (ABE) assay using anti-GluA1 antibodies. Decreased levels of GluA1 palmitoylation were found in the amygdala slices from GluA1C811S mice (42.2 ± 11.4%, compared to wt control, *n* = 3, respectively; *p* < 0.01; *t*-test; Supplementary Fig. 7A). The residual signals likely represented the palmitoylation at another site, Cys585 on transmembrane domain (TMD) 2, which regulates the AMPA receptor localization in the Golgi apparatus, not synaptic membrane trafficking, and is intact in GluA1C811S mice [26, 31]. Along with that, GluA1 expression in postsynaptic density (PSD) fraction was enhanced in the amygdala of GluA1C811S mice (148.0 ± 6.1%, compared to wt control, *n* = 4, respectively; *p* < 0.01; *t* test; Supplementary Fig. 7B), whereas total GluA1 protein amount showed similar level with wt mice (Supplementary Fig. 7B).

## Discussion

Although the AMPA receptor GluA1 subunit is widely expressed in excitatory synapses throughout the brain, we found the influence of its C811S non-palmitoylation mutation on behavior after intense shock to be quite limited. Our comprehensive behavioral battery tests show that general health, sensitivity, locomotion, sociability related to autism spectrum disorder, depression-related behaviors, and spatial learning and memory are unaffected by the C811S mutation in GluA1. On the other hand, increased formation (Fig. 2A) and poor extinction (Fig. 2C) of contextual fear and enhancement of fear generalization (Fig. 2D) were observed in GluA1C811S mice despite their normal anxiety-like behaviors at the basal level (Fig. 1). In contrast to contextual fear, cued fear was not influenced by GluA1 palmitoylation (Fig. 2A, D). Previous behavioral study revealed that both contextual and auditory cued fear conditioning are especially resistant to test order [38]. Decreased palmitoylation of GluA1 in the amygdala of GluA1C811S mice corresponds exactly to our behavioral results (Supplementary Fig. 7A). Enormous cellular heterogeneity is known in complicated structure of amygdala, which comprises 13 or more subnuclei including the basal and lateral subregions, known as the BLA [56–58]. In addition to amygdala, fear memory is regulated by its excitatory and inhibitory connections among PFC, hippocampus, and thalamus [53, 59–61]. In the current study, we performed slice patch-clamp recordings from BLA pyramidal neurons and found hyperexcitability of glutamatergic synapses in the BLA of GluA1C811S mice (Fig. 3C). The BLA is mainly involved in the fear extinction pathway that originates from the infralimbic cortex in the medial PFC [62–65]. These augmentations suggest that contextual fear extinction is notably regulated by GluA1 palmitoylation in the postsynapses in BLA [56]. In contrast, we have previously shown that there is no significant difference in the ratio of AMPA receptor- to NMDA receptor-mediated synaptic currents in hippocampal CA1 pyramidal neurons between wt and GluA1C811S mice [34]. Our results revealed a specific role of the AMPA receptor palmitoylation-mediated reduction of contextual fear in BLA. Moreover, biochemical results of reduced GluA1 palmitoylation and increased synaptic expression of GluA1 in PSD of the amygdala (Supplementary Fig. 7A, B), which should enhance AMPA receptor synaptic retention as well as synaptic plasticity [26, 27, 32, 34], explain behavioral alterations well. Cue (tone)-dependent (auditory) fear conditioning is mediated by the potentiation of glutamatergic synaptic transmission in the lateral amygdala [66, 67]. Fear generalization is mediated by coordinated actions of the PFC, hippocampus, amygdala, and thalamus [52]. Freezing response was increased in palmitoylation-deficient GluA1C811S mice even by transferring from home cage to extraordinary chamber without being affected by tone cue. This phenomenon suggests that mice choose safer way to avoid potential threats in AMPA receptor palmitoylation-dependent manner, which seems to be related to primate fear generalization based on negative experience [51].

PTSD is triggered by brief re-exposure to sights, sounds, smells, or thoughts which remind patient of the traumatic event. Previous studies showed that PTSD involves an impairment of fear extinction [4, 68]. Actual or imaginal prolonged exposure to traumatic cues is employed to induce habituation in the psychotherapy for PTSD, known as exposure therapy and cognitive behavioral therapy [69–71]. Epidemiological investigations show that females are more likely to be affected by PTSD than males [72–74]. Influence of AMPA receptor palmitoylation deficiency on long-lasting fear, which was experimentally observed even in palmitoylation-deficient male mice as mentioned above, may be more serious in female. The sexual differences in AMPA receptor palmitoylation-related fear should be further investigated in the future. Even though GluA1C811S mice were repeatedly re-exposed to the contextual environments, such extinction training had little effect on contextual fear in the case of late-start intervention (Fig. 2C). These results indicate that palmitoylation of the AMPA receptor is indispensable to reduce contextual fear at a very early stage. Extinction training just after fear memory formation is thought to be crucial for effective treatment of human PTSD [70, 71]. In the current study, similar decreases in freezing rates were observed only in wt, not in GluA1C811S, mice after long exposure every day (Fig. 3A). Consistent with human therapeutic accumulations and our previous reports [54, 55], shorter exposure had little effect (Fig. 3B). Taken together, appropriate regulation of GluA1 palmitoylation in pyramidal neurons in the BLA soon after initial traumatic event is necessary to suppress long-term excessive fear, which may play an important role in preventing PTSD.

## Supplementary information

Supplementary information
