# Supplementary material for: Prolonged contextual fear memory in AMPA receptor palmitoylation-deficient mice
Source: Neuropsychopharmacology. 2022 May 26;47(12):2150–9. doi: 10.1038/s41386-022-01347-9 (PMC9556755; doi:10.1038/s41386-022-01347-9)
Supplement: Supplementary file 1 — Supplementary information [file 41386_2022_1347_MOESM1_ESM.pdf]

Prolonged contextual fear memory in AMPA receptor palmitoylation-deficient mice

Akiko Oota-Ishigaki[1], Keizo Takao[2,3], Daisuke Yamada[4], Masayuki Sekiguchi[4], Masayuki Itoh[4], Yumie Koshidata[2,3], Manabu Abe[5], Rie Natsume[5], Masaki Kaneko[4], Toma Adachi[4], Toshie Kaizuka[4], Nami Suzuki[1], Kenji Sakimura[5], Hiroyuki Okuno[6,7], Keiji Wada[4], Masayoshi Mishina[8], Tsuyoshi Miyakawa[2,9], Takashi Hayashi[1,4]*

[1]Biomedical Research Institute, National Institute of Advanced Industrial Science and Technology (AIST), Tsukuba, Ibaraki 305-8566, Japan
[2]Section of Behavior Patterns, Center for Genetic Analysis of Behavior, National Institute for Physical Sciences (NIPS), Okazaki, Aichi 444-8585, Japan
[3]Life Science Research Center, University of Toyama, Toyama 930-0194, Japan
[4]National Institute of Neuroscience, National Center of Neurology and Psychiatry (NCNP), Kodaira, Tokyo 187-8502, Japan
[5]Department of Cellular Neurobiology, Brain Research Institute, Niigata University, Niigata 951-8585, Japan
[6]Medical Innovation Center/SK Project, Graduate School of Medicine, Kyoto University, Kyoto 606-8507, Japan
[7]Department of Biochemistry and Molecular Biology, Kagoshima University Graduate School of Medical and Dental Sciences, Kagoshima 890-8544, Japan
[8]Brain Science Laboratory, The Research Organization of Science and Technology, Ritsumeikan University, Kusatsu, Shiga 525-8577, Japan
[9]Division of Systems Medical Science, Institute for Comprehensive Medical Science, Fujita Health University, Toyoake, Aichi 470-1192, Japan

* Correspondence should be addressed to T. H., Central6 (6-10), 1-1-1 Higashi, AIST, Tsukuba, Ibaraki 305-8566, Japan
Tel: +81-29-861-8397
E-mail: takashi.hayashi@aist.go.jp

Supplementary information

Materials and Methods

Comprehensive behavioral battery tests

Neurological Screen

Neurological screening was performed as previously described [75]. The righting, whisker touch, and ear twitch reflexes were evaluated. A number of physical features, including the presence of whiskers or bald hair patches, were also recorded.

Neuromuscular examination

Neuromuscular strength was examined using the grip strength and wire hang tests as previously described [44]. A grip strength meter (O’Hara & Co., Tokyo, Japan) was used to assess forelimb grip strength. Each mouse was tested three times, and the greatest value was used for statistical analysis. In the wire hang test, a box (21.5 × 22 × 23 cm^3) with a wire mesh grid (10 × 10 cm^2) on top (O’Hara & Co.) was used. Latency to fall was recorded, with a 60-s cutoff time.

Acoustic startle response and prepulse inhibition test

A mouse restrained in a cylinder was placed in the chamber of a startle reflex measurement system (O'Hara & Co.) with 70-dB background white noise. After 10 min, the mouse’s startle response to a startle stimulus (40 ms of 110- or 120-dB white noise) was measured by a motion sensor for 140 ms (temporal resolution, 1 ms). A test session was a random sequence of four trials with a prepulse stimulus (40 ms of 74- or 78-dB white noise 100 ms prior to a startle stimulus) and two trials without a stimulus. Six blocks of six trial types were presented in a pseudorandom order with an average inter-trial interval of 15 s.

Hot plate test

The hot plate test was used to evaluate sensitivity to a painful stimulus. Mice were placed on a 55.0 (± 0.3) °C hot plate (Columbus Instruments, Columbus, OH, USA), and latency to the first hind paw response was recorded. The hind paw response was defined as either a foot shake or a paw lick.

Gait analysis

Automated gait analysis on a treadmill was conducted using DigiGait (Mouse Specifics Inc., Framingham, MA, USA). Each mouse was forced to walk on a treadmill moving at 24 cm/s, while the mouse body movement and paw footprints were recorded at 150 frames/s with a CCD camera underneath the treadmill. Multiple quantitative parameters (length, width, and timing of the strides, paw angle, etc.) were extracted from the time-lapse images with bundled software.

Rotarod test

The rotarod test was conducted using an accelerating rotarod apparatus (UGO Basile, Comerio-Varese, Italy), as previously described [46]. Mice were placed on a rotating drum and their latency to fall was recorded with a 300-s cutoff. The speed of the drum was accelerated from 4 to 40 rpm over a 5-min period. The mice were given three trials per day for 2 consecutive days.

Beam test

The beam test was performed as previously reported [76] to measure the ability of mice to traverse a narrow beam to reach an escape box. The 100 cm-long beam, with a rough painted surface, consisted of iron (2.8-cm or 1.0-cm width in diameter) placed horizontally 50 cm above the platform surface. The mice were trained to traverse the length of the wide beam, which led to the escape box. Motor coordination was quantitatively assessed by trial performance, moving speed, and the number of slips along the beam. After three trials using the wide beam were conducted in 1 day, three additional trials with a narrow beam were conducted the following day. Before the first trial of the day, the mice were allowed to habituate to the beam. The increments in moving speed and decrements in the number of slips were quantified as indices of motor coordination using ImageBT software.

Light/dark transition test

A light/dark transition test was conducted as previously reported [77]. Mice were placed in the dark side and allowed to move freely between two chambers with an open door in between for 10 min. The total number of transitions between the chambers, time spent on each side, first latency to enter the light side, and distance traveled in each chamber were recorded.

Open field test

Locomotor activity was measured in an open field apparatus (40 × 40 × 30 cm^3; Accuscan Instruments, Columbus, OH, USA) as described earlier [48]. Total distance traveled, vertical activity (rearing measured by counting the number of photobeam interruptions), time spent in the center (20 × 20 cm^2) of the open field area, and stereotypic counts were recorded using a VersaMax system (Accuscan Instruments). Data were collected for 120 min.

Elevated plus maze test

The elevated plus maze test was conducted as described previously [78]. Mouse behavior was recorded during a 10-min test period. The number of entries into and the time spent in the open and enclosed arms were recorded.

Novelty-induced hypophagia

Anxiety-related behavior was evaluated with a novelty-induced hypophagia test. In the training session, mice were group-housed (four mice per cage) and exposed for 30 min to 25% sweetened condensed milk diluted with water from sippers attached to a 5-ml pipette in each cage on 3 consecutive days. After the first 24 h of the training session, the mice were divided into two groups: a home cage group and a novel environment group. For home cage testing, the mice were first removed from their home cage and placed in another cage. After 30 min, one mouse was returned to each home cage in a dark environment (50 lx), and the latency to drink and volume consumed were recorded every 5 min for 30 min. In the novel environment group, each mouse was placed in a new cage fitted with a white acryl flooring under bright lighting (1000 lx), and the latency to drink and volume consumed were recorded every 5 min for 30 min.

Social interaction test in a novel environment (one-chamber)

The social interaction test was conducted as previously described [45]. Two mice of identical genotypes that were previously housed in different cages were placed in a box together (40 × 40 × 30 cm^3) and allowed to explore the box freely for 10 min. Images were captured at 1 frame per second, and the distance traveled between two successive frames was calculated for each mouse. The average distance traveled of both mice was analyzed. If the two mice had contact with each other and if the distance traveled by either mouse was longer than 10 cm, their behavior was recorded as “active contact”.

Crawley’s sociability and preference for social novelty test

The test for sociability and preference for social novelty was conducted as previously described [79,80]. One day before testing, the subject mice were individually placed in the middle chamber of three and allowed to freely explore the entire apparatus for 10 min. In the sociability test, an unfamiliar C57BL/6J male mouse (stranger 1) that had no prior contact with the subject mouse was placed in one of the side chambers. The subject mouse was first placed in the middle chamber and allowed to explore the three chambers for 10 min. At the end of the first 10 min, each mouse was tested in a second 10-min session to quantify social preference for a new stranger. A second, unfamiliar mouse was placed in the chamber that had been empty during the first 10-min session. The second stranger was enclosed in an identical small wire cage. The test mouse had a choice between the first, already-investigated unfamiliar mouse (stranger 1) and the novel unfamiliar mouse (stranger 2). The stranger mice used in this experiment were 8- to 12-week-old C57BL/6J male mice, not littermates that had previously been habituated to placement in the small circular wire cage.

Social interaction test in home cage

Social interaction monitoring in the home cage was conducted as previously described [81,82]. The system comprised a home cage (29 × 18 × 12 cm^3) and a filtered cage top, separated by a 13 cm-high metal stand containing an infrared video camera attached to the top of the stand. Two mice of the same inbred strain that had been housed separately were placed together in a home cage. Their social behavior was then monitored for 1 week. The output from the video camera was fed into a Macintosh computer. Images from each cage were captured at a rate of one frame per second. Social interaction was measured by counting the number of particles detected in each frame: two particles indicated that the mice were not in contact with each other, and one particle (i.e., the tracking software could not distinguish two separate bodies) indicated contact between the two mice. We also measured locomotor activity during these experiments by quantifying the number of pixels that changed between each pair of successive frames. Analysis was performed automatically using Image HA software.

Barnes maze test

The Barnes maze test was conducted on a white circular platform, 1.0 m in diameter, with 12 holes equally spaced around the perimeter (O’Hara & Co.), essentially as previously described [47]. Prior to beginning the test, the mice completed one habituation trial to familiarize themselves with the maze and the existence of the escape box. In the test, the trial ended when the mouse entered the escape box or after 5 min elapsed. Three trials per

day were conducted for 4 consecutive days. On day 5, the mice underwent a probe trial conducted without the escape box for 3 min to confirm that the spatial task was carried out based on navigation by distal environmental room cues. The mice were then left undisturbed until the next probe trial. On day 35, the mice underwent a probe trial to check remote memory. A single training trial was conducted immediately after each trial. For the reversal task, the target was moved to a new position opposite to the original after the training trials for another 5 consecutive days. The mice were then trained for 3 consecutive days and underwent another probe trial.

T-maze test

The spontaneous alternation task was conducted using an automated T-maze apparatus (O'Hara & Co.), as previously described [83].

Non-associative place recognition test

To assess the mice's pattern separation abilities, a non-associative place recognition test was performed as previously described [84]. The square-type (S) chamber consisted of a transparent acrylic board (width × depth × height: 30 × 30 × 30 cm^3), and the chamber floors were covered by a stainless-steel wire mesh (aperture size, 7.5 × 7.5 mm^2) with a diameter of 1 mm. The circle-type (C) chamber was cylindrical (diameter × height: 170 × 270 mm^2) and had a white opaque acrylic board wall and floor.

Mice were randomly assigned to four groups: two same-combination groups (S-S, C-C), and two different-combination groups (S-C, C-S). During the learning session, mice were placed in one of the chambers (S or C). After 6 min, the mice were returned to their home cages. After 24 h, the test session was conducted. During the testing session, the mice were placed into the already-experienced or the novel chamber for 3 min. The chamber was set at the same position in the soundproof room for the behavior experiments. Distance traveled was measured as an index of motility using ImageOF software.

Porsolt forced swim test

The apparatus consisted of four plastic cylinders (20 cm height × 10 cm diameter). The cylinders were filled with water (23 °C) up to a height of 7.5 cm. Mice were placed into the cylinders, and their behavior was recorded over a 10-min test period. Data acquisition and analysis were performed automatically using Image PS software. Distance traveled was measured using Image OF software and stored image files.

Tail suspension test

The tail suspension test was performed over a 10-min test duration. Mice were suspended 30 cm above the floor in a visually isolated area with adhesive tape placed 1 cm from the tip of the tail, and their behavior was recorded over a 10-min test period. Data acquisition and analysis were performed automatically using Image TS software.

Contextual and cued fear conditioning

Contextual and cued fear conditioning tests were conducted as described previously [45,85] at the NIPS, as shown in Fig. 2. A 55-dB white noise, which served as the conditioned stimulus (CS), was played for 30 s. During the last 2 s of the tone, a footshock (0.3 mA) was delivered as the unconditioned stimulus (US). Each mouse received three CS–US pairings with a 2-min interstimulus interval. Contextual testing was conducted 24 h after conditioning. The mice were monitored for freezing for 5 min in the same chamber and then returned to their home cages. Cued testing in an altered context was conducted 3 h after context testing using a triangular box (35 × 35 × 40 cm^3) made of white opaque Plexiglas, which was located in a different room. Freezing behavior was assessed during a 3-min free exploration, followed by a 3-min presentation of the tone.

Contextual fear conditioning and extinction tests were performed as described previously [54,55] at the NCNP, as shown in Fig. 3. Briefly, naïve mice were acclimated to the test room for 1 h before fear conditioning and placed in a conditioning chamber (20 × 20 cm^2 floor, 35 cm high; Muromachi Kikai, Co., LTD. Tokyo, Japan), permitted to explore the chamber for 150 s, and conditioned with a double electrical footshock (0.8 mA, 2-s duration, 1-min interval). After conditioning, the mice were kept in the conditioning chamber for an additional 30 s before returning to their home cages. After 24 h, the mice were re-exposed to the same chamber for 10 min without footshock to measure the freezing response. For the extinction test, the exposure session was repeated for 5 consecutive days.

Electrophysiology

Preparation of brain slices and whole-cell recordings were performed as described previously with minor modifications [34,35]. Briefly, naïve mice at postnatal days 26–38 were anesthetized with sevoflurane and brains were quickly removed (P27–38 for wt, P26–37 for GluA1C811S). Coronal slices containing the basolateral amygdala (BLA) (300 µm) were prepared using a linear slicer Pro 7 (Dosaka EM, Kyoto, Japan) in an ice-cold solution (in mM: 100 NMDG-Cl, 2.5 KCl, 1.25 NaH_2PO_4, 30 $NaHCO_3$, 20 HEPES, 25 glucose, 2 thiourea, 5 Na-ascorbate, 2.5 Na-pyruvate, 1 N-acetyl-L-cysteine (NAC), 0.5 $CaCl_2$, and 10 $MgSO_4$). After sectioning, the slices were recovered with the cutting

medium for 10 min at 32 °C, and then stored for at least 1 h at room temperature in a solution of (in mM) 100 NaCl, 2.5 KCl, 1.25 NaH_2PO_4, 30 $NaHCO_3$, 20 HEPES, 25 glucose, 2 thiourea, 5 Na-ascorbate, 2.5 Na-pyruvate, 1 NAC, 2 $CaCl_2$ and 2 $MgSO_4$. Each slice was then transferred to the recording chamber and perfused (at approximately 3 ml/min) with artificial cerebrospinal fluid (ACSF) (in mM: 125 NaCl, 2.5 KCl, 26 $NaHCO_3$, 1.25 NaH_2PO_4, 4 $CaCl_2$, 4 $MgSO_4$, and 10 glucose) at 28–32 °C. All external solutions were equilibrated with 95% O_2 and 5% CO_2 (pH 7.4). Electrophysiological signals were acquired using a MultiClamp 700B, Digidata 1440, and pClamp 10 (Molecular Devices, Sunnyvale, CA, USA). The liquid junction potential and series resistance were left uncompensated. Series resistance was monitored frequently during recordings, and neurons showing >30 MΩ and a large drift (20%) in series resistance were excluded from the analysis. For voltage-clamp recordings, patch pipettes were filled with Cs^+-based intracellular solution (in mM: 135 $CsMeSO_3$, 5.0 TEA-Cl, 1 $MgCl_2$, 0.2 EGTA, 3.0 Mg-ATP, 0.3 Na-GTP, 10 Na-phosphocreatine, 2 QX314, and 10 HEPES (pH 7.2)) with a resistance of 4–6 MΩ. A bipolar stimulating electrode (FHC) was placed near the patched cells (<50 µm) to evoke baseline AMPAR-EPSC of -200 to -400 pA at -70 mV, and the holding potential was then switched to +40 mV to measure the NMDAR-current. For measurement of AMPA/NMDA ratios, we used a bath solution containing 100 µM pictrotoxin. AMPA/NMDA ratios were calculated as the ratio of the peak AMPAR-current at -70 mV to the NMDAR-current 80 ms after stimulus onset at +40 mV.

Biochemical analysis

Palmitoylation of GluA1 protein was assessed using the acyl-biotinyl exchange (ABE) method as described previously [34,86]. Briefly, the amygdala slices were prepared from 11-17 weeks old naïve wt and GluA1C811S mutant mice. Dissected slices (~15 mg/slice) were directly denatured in 400 µl lysis buffer (25 mM HEPES (pH 7.4), 150 mM NaCl, 2% SDS, 5 mM EDTA, protease inhibitor cocktail (Roche), 20 mM methyl methanethiosulfonate (MMTS) to block free thiols), and acetone precipitation was used to move between steps. Because of slight variation in individual size and weight of prepared amygdala slices, five slices from each genotype were combined after lysis. Following lysis, excess MMTS was removed by acetone precipitation, and pellets were resuspended in a buffer containing 4% (w/v) SDS buffer (4SB). Equally divided samples were diluted and incubated for 1 h in either 0.7 M hydroxylamine (NH_2OH, pH 7.4), to cleave thioester bonds, or 50 mM Tris (pH 7.4). After acetone precipitation to remove hydroxylamine, or Tris, pellets were resuspended in 4SB, and incubated for 1 h in 50 mM Tris (pH 7.4) containing 0.2 mM sulfhydryl-reactive biotin-HPDP (Cayman Chemical,

Ann Arbor, MI, USA) at room temperature. To remove unreacted HPDP-biotin, acetone precipitation was performed and the pellets were resuspended in 4SB. SDS was then diluted to 0.1% (w/v) and biotinylated proteins in the samples were affinity-purified using Streptavidin Mag Sepharose (GE Healthcare, Tokyo, Japan). 40 μl SDS sample buffer was used to cleave HPDP-biotin and release purified proteins from the beads. The total proteins released in the supernatant were denatured in SDS sample buffer. Preparation of postsynaptic density (PSD) fraction was performed as previously reported [87]. Samples were then separated by SDS-PAGE, followed by western blotting with anti-GluA1 (ab31232, Abcam, Cambridge, UK), anti-GluA2 (cst13607, Cell Signaling Technology, Danvers, MA, USA) or anti-GluN1 (cst5704, Cell Signaling Technology) antibodies. Five percent of total amygdala lysates from wt and GluA1C811S mutant were loaded to confirm protein expression level (shown as "lysate" in Supplementary Figures 7A and 7B, left panels). Reactive bands were visualized with the ECL Prime Western Blotting Detection System, and chemiluminescent images were acquired using the ImageQuant LAS 4000 mini imager (GE Healthcare). The palmitoylation ratio was estimated by comparing the intensity of each band using ImageJ: palmitoylated GluA1 amounts (NH_2OH (+)) to total GluA1 amounts in the amygdala slice lysates (lysate).

References

34 Itoh M, Yamashita M, Kaneko M, Okuno H, Abe M, Yamazaki M, et al. Deficiency of AMPA receptor-palmitoylation aggravates seizure susceptibility. J Neurosci. 2018;38:10220–35.

35 Itoh M, Okuno H, Yamada D, Yamashita M, Abe M, Natsume R, et al. Perturbed expression pattern of the immediate early gene Arc in the dentate gyrus of GluA1 C-terminal palmitoylation-deficient mice. Neuropsychopharmacol Rep. 2019;39:61–66.

44 Arron JR, Winslow MM, Polleri A, Chang CP, Wu H, Gao X, et al. NFAT dysregulation by increased dosage of DSCR1 and DYRK1A on chromosome 21. Nature. 2006;441:595–600.

45 Ihara M, Yamasaki N, Hagiwara A, Tanigaki A, Kitano A, Hikawa R, et al. Sept4, a component of presynaptic scaffold and Lewy bodies, is required for the suppression of alpha-synuclein neurotoxicity. Neuron. 2007;53:519–33.

46 Miyakawa T, Yamada M, Duttaroy A, Wess J. Hyperactivity and intact hippocampus-dependent learning in mice lacking the M1 muscarinic acetylcholine receptor. J Neurosci. 2001;21:5239–50.

47 Miyakawa T, Yared E, Pak JH, Huang FL, Huang KP, Crawley JN. Neurogranin null mutant mice display performance deficits on spatial learning tasks with anxiety related components. Hippocampus. 2001;11:763–75.

54 Yamada D, Wada K, Sekiguchi M. Facilitating actions of an AMPA receptor potentiator upon extinction of contextually conditioned fear response in stressed mice. Neurosci Lett. 2011;488:242–6.

55 Yamada D, Zushida K, Wada K, Sekiguchi M. Pharmacological discrimination of extinction and reconsolidation of contextual fear memory by a potentiator of AMPA receptors. Neuropsychopharmacology. 2009;34:2574–84.

75 Born TL, Smith DE, Garka KE, Renshaw BR, Bertles JS, Sims JE. Identification and characterization of two members of a novel class of the interleukin-1 receptor (IL-1R) family. Delineation of a new class of IL-1R-related proteins based on signaling. J Biol Chem. 2000;275:29946–54.

76 Ohno M, Hiraoka Y, Matsuoka T, Tomimoto H, Takao K, Miyakawa T, et al. Nardilysin regulates axonal maturation and myelination in the central and peripheral nervous system. Nat Neurosci. 2009;12:1506–13.

77 Takao K, Miyakawa T. Light/dark transition test for mice. J Vis Exp. 2006:104.

78 Komada M, Takao K, Miyakawa T. Elevated plus maze for mice. J Vis Exp.

2008:1088.
79 Moy SS, Nadler JJ, Perez A, Barbaro RP, Johns JM, Magnuson TR, et al. Sociability and preference for social novelty in five inbred strains: an approach to assess autistic-like behavior in mice. Genes Brain Behav. 2004;3:287–302.
80 Tanda K, Nishi A, Matsuo N, Nakanishi K, Yamasaki N, Sugimoto T, et al. Abnormal social behavior, hyperactivity, impaired remote spatial memory, and increased D1-mediated dopaminergic signaling in neuronal nitric oxide synthase knockout mice. Mol Brain. 2009;2:19.
81 Carrie A, Jun L, Bienvenu T, Vinet MC, McDonell N, Couvert P, et al. A new member of the IL-1 receptor family highly expressed in hippocampus and involved in X-linked mental retardation. Nat Genet. 1999;23:25–31.
82 Miyakawa T, Leiter LM, Gerber DJ, Gainetdinov RR, Sotnikova TD, Zeng H, et al. Conditional calcineurin knockout mice exhibit multiple abnormal behaviors related to schizophrenia. Proc Natl Acad Sci U S A. 2003;100:8987–92.
83 Shoji H, Hagihara H, Takao K, Hattori S, Miyakawa T. T-maze forced alternation and left-right discrimination tasks for assessing working and reference memory in mice. J Vis Exp. 2012:3300.
84 Kitamura T, Okubo-Suzuki R, Takashima N, Murayama A, Hino T, Nishizono H, et al. Hippocampal function is not required for the precision of remote place memory. Mol Brain. 2012;5:5.
85 Shoji H, Takao K, Hattori S, Miyakawa T. Contextual and cued fear conditioning test using a video analyzing system in mice. J Vis Exp. 2014:50871.
86 Wan J, Roth AF, Bailey AO, Davis NG. Palmitoylated proteins: purification and identification. Nat Protoc. 2007;2:1573–84.
87 Villasana LE, Klann E, Tejada-Simon MV. Rapid isolation of synaptoneurosomes and postsynaptic densities from adult mouse hippocampus. J Neurosci Methods. 2006;158:30–6.

Figure legends

Supplementary Figure 1. Normal locomotor ability in GluA1C811S mutant mice.

A) Rotarod test: The time that the mice remained on the rotating rod before falling was measured. The *p*-value indicates the genotype effect in two-way repeated measures ANOVA.

B) Beam test: The ability of mice to traverse a beam to reach an escape box was assessed. Moving speed (left), number of moving episodes (middle), and number of slips (right) on the beam are shown. The *p*-value indicates the genotype effect in two-way repeated measures ANOVA.

C) Gait analysis: The front paw (top) and hind paw (bottom) stride or stance duration of each step, stance width, stride length, and step or paw angle are shown.

All data are expressed as mean ± SEM. The *p*-values indicate genotype effects.

Supplementary Figure 2. Normal anxiety-like behaviors in GluA1C811S mutant mice.

A) Open field test: count of vertical activity (A1), and count of stereotypic behavior (A2).

Supplementary Figure 3. Normal social behaviors in GluA1C811S mutant mice.

A) Social interaction in a novel environment: total duration of contacts (A1), number of contacts (A2), total duration of active contacts (A3), mean duration of each contact (A4), and total distance traveled (A5).

B) Three-chamber sociability and social novelty preference tests: Sociability test (B1). Percent time spent around the cage containing a stranger (left, shown as [Stranger (Str.)/Stranger (Str.)+Empty (Emp.)]) and distance traveled (right) are shown. Social novelty preference test (B2). Percent time spent around the cage containing a stranger (stranger 2) (left, shown as [Stranger (Str.)/Stranger (Str.)+Familiar (Fam.)]) and distance traveled (right) are shown.

C) Social interaction in the home cage: mean number of particles detected (top) and activity levels (bottom) were recorded over 7 days (C1). Diurnal oscillation of the locomotor activity in the home cage is shown as an averaged value over 3 days (C2). The *p*-values indicate the genotype effect in a two-way repeated measures ANOVA.

All data are expressed as mean ± SEM. The *p*-values indicate genotype effects.

Supplementary Figure 4: Normal depression-related behaviors in GluA1C811S mutant mice.

Depression-related behaviors in GluA1C811S mice were assessed using the forced swim and tail suspension tests.

A) Porsolt forced swim test: immobility time in each block (top, %) and distance traveled in each block (bottom, cm) were recorded.
B) Tail suspension test: immobility time (%) was recorded in each block.

All data are expressed as mean ± SEM. The *p*-values indicate genotype effects.

Supplementary Figure 5. Normal learning and memory in GluA1C811S mutant mice.

A) Barnes circular maze: number of errors before reaching the target hole and latency to reach the target hole during the training session (A1). Data were analyzed using two-way repeated measures ANOVA. Data are presented as the means of three trials. The *p*-values indicate the genotype effect in two-way repeated measures ANOVA. The time spent around each hole in the probe trial was conducted 24 h (A2) and 30 d (A3) after the last training session. Reversal training in the Barnes maze test (A4). Number of errors before reaching the target hole (left) and latency to reach the target hole during the training session (right). The time spent around each hole in the probe trial was conducted 1 d after the last training (A5, shown as (Spent Time (ST) in Target)/(ST in Target+ST in Angle ±30° (A±30))). Time in 180 (angle ±180°) indicates the exploration of the target before reversal.
B) The percentage of correct responses in the T-maze spontaneous alteration task.
C) Pattern separation: Distance traveled in the first 3 min during the learning or testing session is shown. Each genotype was tested with the same combinations (left, S-S and C-C) and the different combinations (right, S-C and C-S, marked by dot or slash) in the non-associative place recognition test. The *p*-values indicate the effect of the combination in the paired-*t* test.

wt (white circle or bar) and C811S (black circle or bar) mice.

Supplementary Figure 6. Cued fear memory in GluA1C811S mutant mice.

Prolonged cue exposure at 47 days after conditioning. Bold line represents tone. All data are expressed as mean ± SEM. The *p*-values indicate genotype effects.

Supplementary Figure 7. Reduced palmitoylation at Cys811 and enhanced synaptic expression of GluA1 in GluA1C811S mutant mice.

A) GluA1 palmitoylation in the amygdala, detected by ABE assay. Representative blots of protein expression (lysate) and palmitoylation (NH_2OH (+)) in the amygdala lysate

prepared from wild-type (wt) and GluA1C811S mice showed that palmitoylation of GluA1 was reduced in GluA1C811S mice (left). Experiments were performed in triplicate (*n* = 3, amygdala slices from five mice each), and palmitoylation levels were normalized to wt mice (right).

B) Total and synaptic protein expression of AMPA receptor subunits GluA1 and GluA2 and the NMDA receptor essential subunit GluN1 in the amygdala. Representative blots of protein expression in total cell lysate and postsynaptic density (PSD) fraction of amygdala prepared from wild-type (wt) or GluA1C811S mice are shown (top). Synaptic GluA1 expression was increased in GluA1C811S mice. Protein expression levels were normalized to wt mice (bottom, *n* = 4, amygdala slices).

wt (white bar) and C811S (black bar) mice. Error bars represent SEM. **$p < 0.01$, *t*-test.

Oota-Ishigaki et al. Supplementary Fig. 1

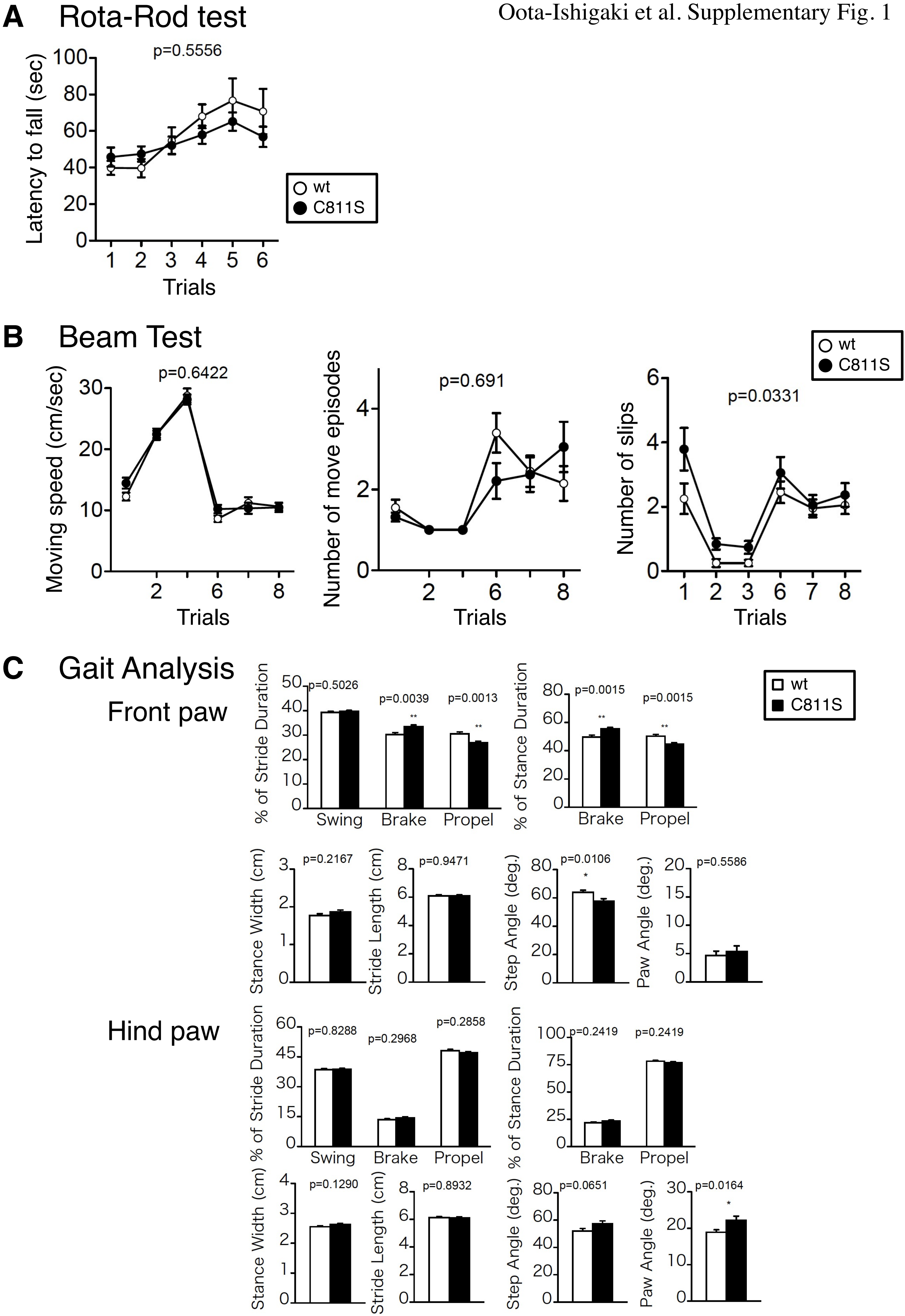

A Open Field test

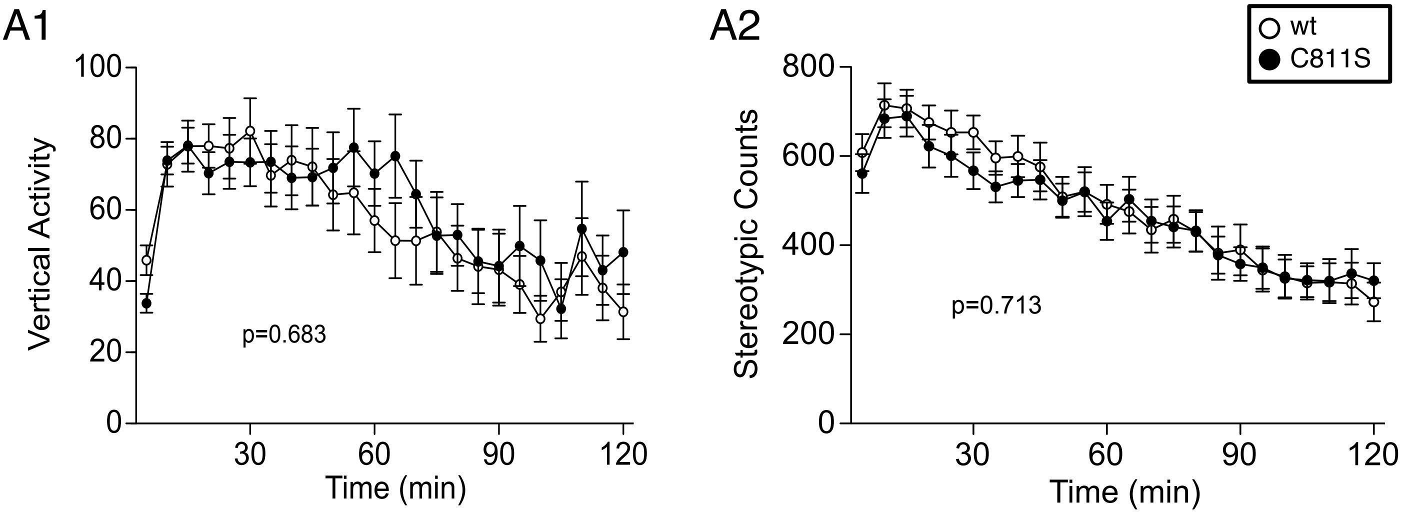

Oota-Ishigaki et al. Supplementary Fig. 2

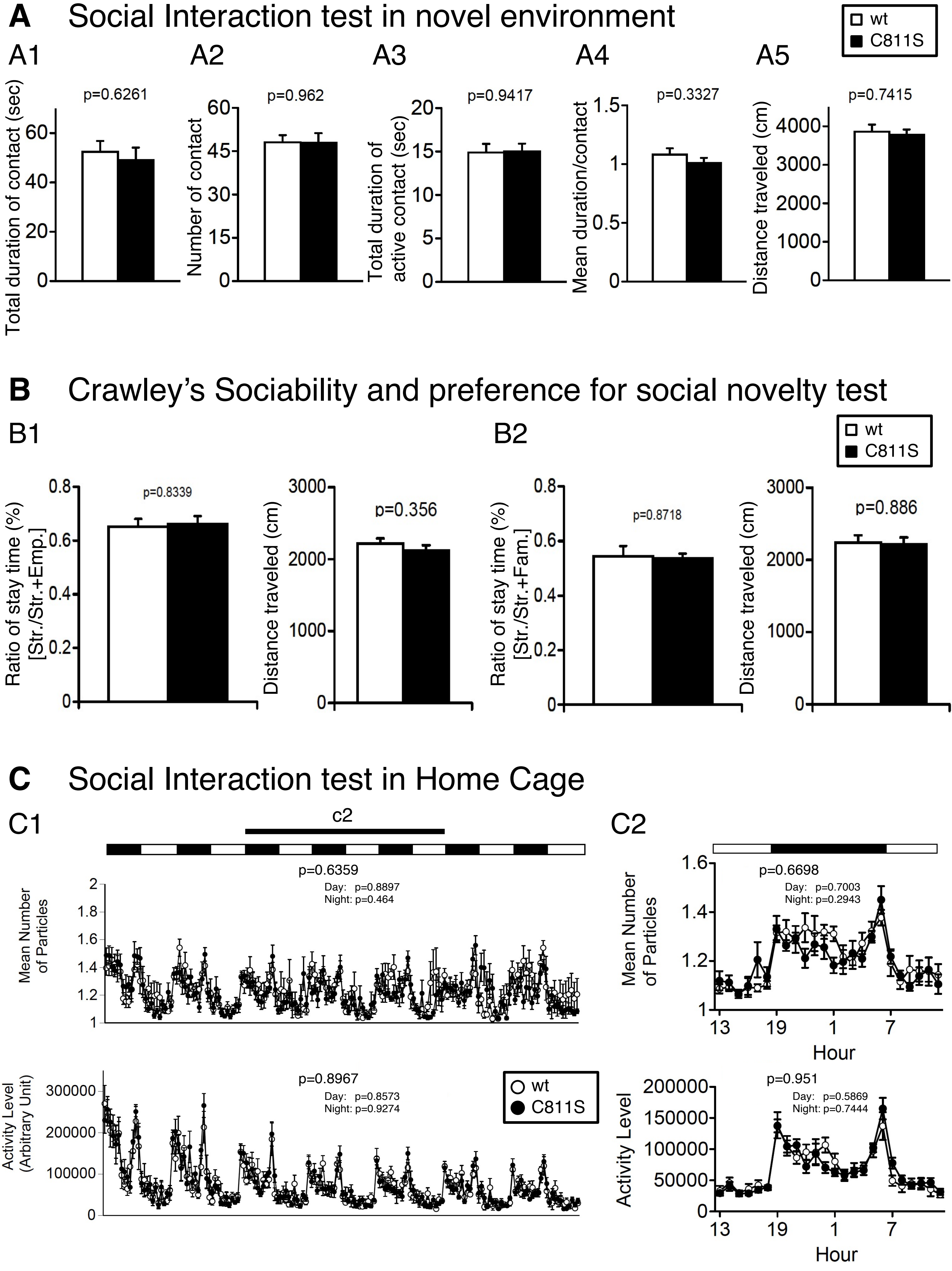

Oota-Ishigaki et al. Supplementary Fig. 3

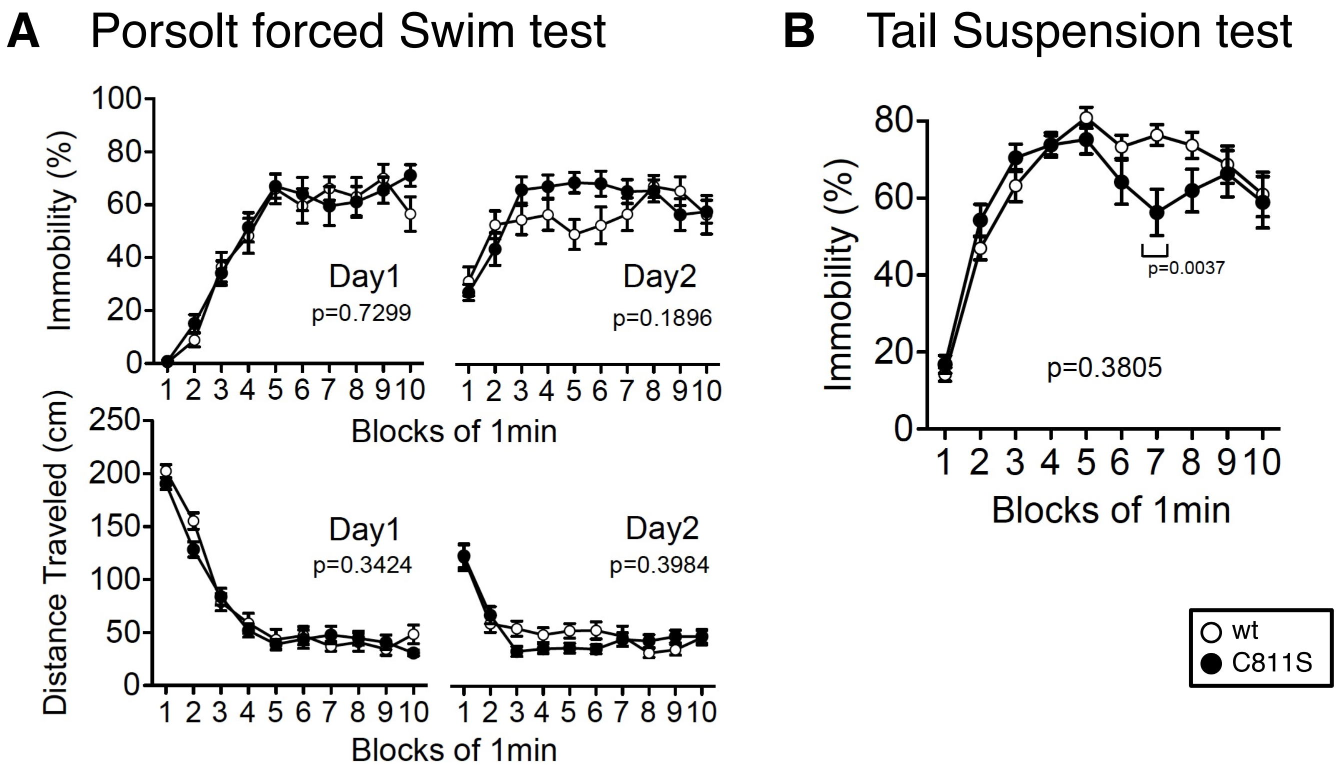

Oota-Ishigaki et al. Supplementary Fig. 4

A Barnes Maze

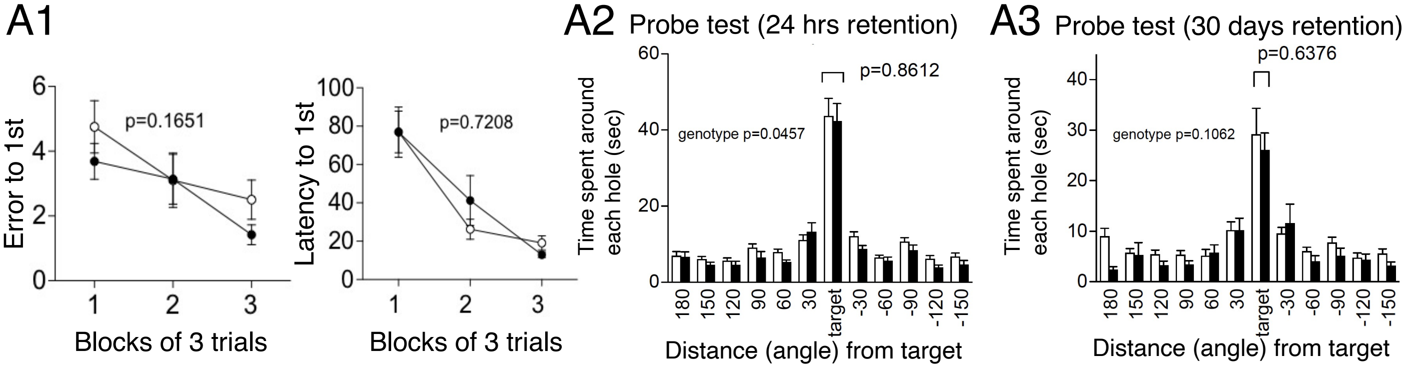

Barnes Maze reversal task

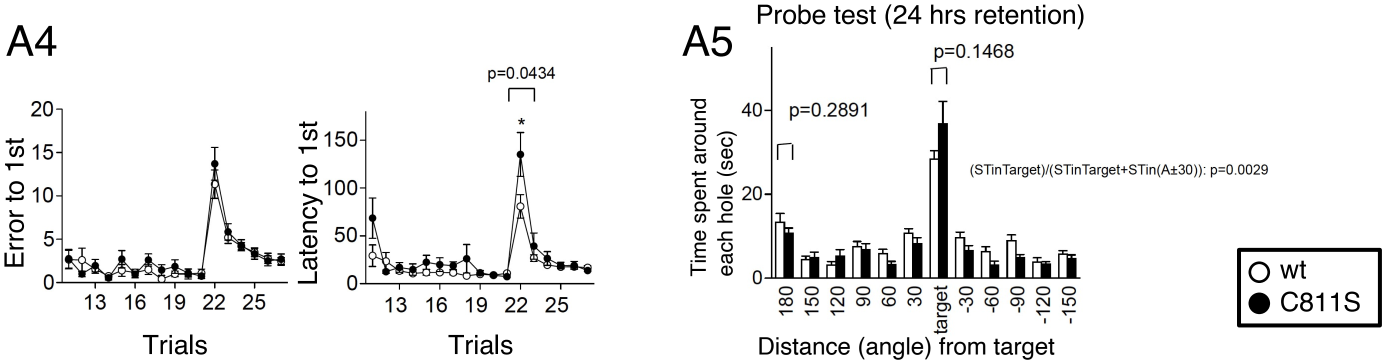

day 0	habituation
day 1	training 01, 02
day 2	training 03, 04, 05
day 3	training 06, 07, 08
day 4	training 09
day 5	Probe test 1, training 10

day 35	Probe test 2, training 11
day 36	training 12, 13
day 37	training 14, 15
day 38	training 16, 17
day 39	
day 40	
day 41	training 18, 19
day 42	training 20, 21
day 43	training 22, 23, reversal training 1, 2
day 44	training 24, 25, reversal training 3, 4
day 45	training 26, 27, reversal training 5, 6
day 46	Probe test 3, training 28, reversal training 7
day 47	training 29, reversal training 8
day 48	training 30, 31
day 49	training 32, 33
day 50	training 34, 35

B T Maze

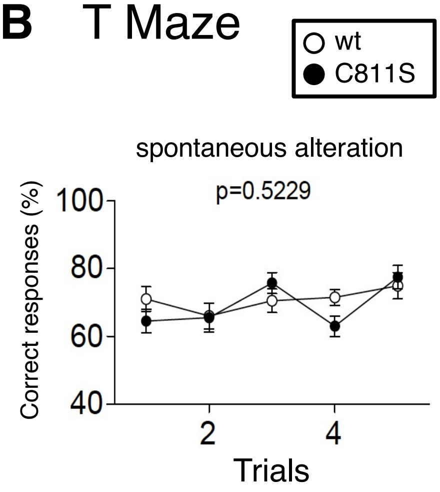

C Pattern Separation

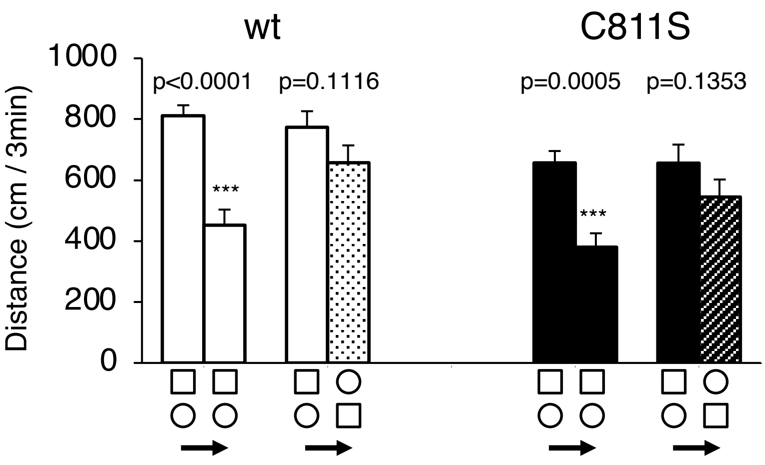

Cued Fear memory

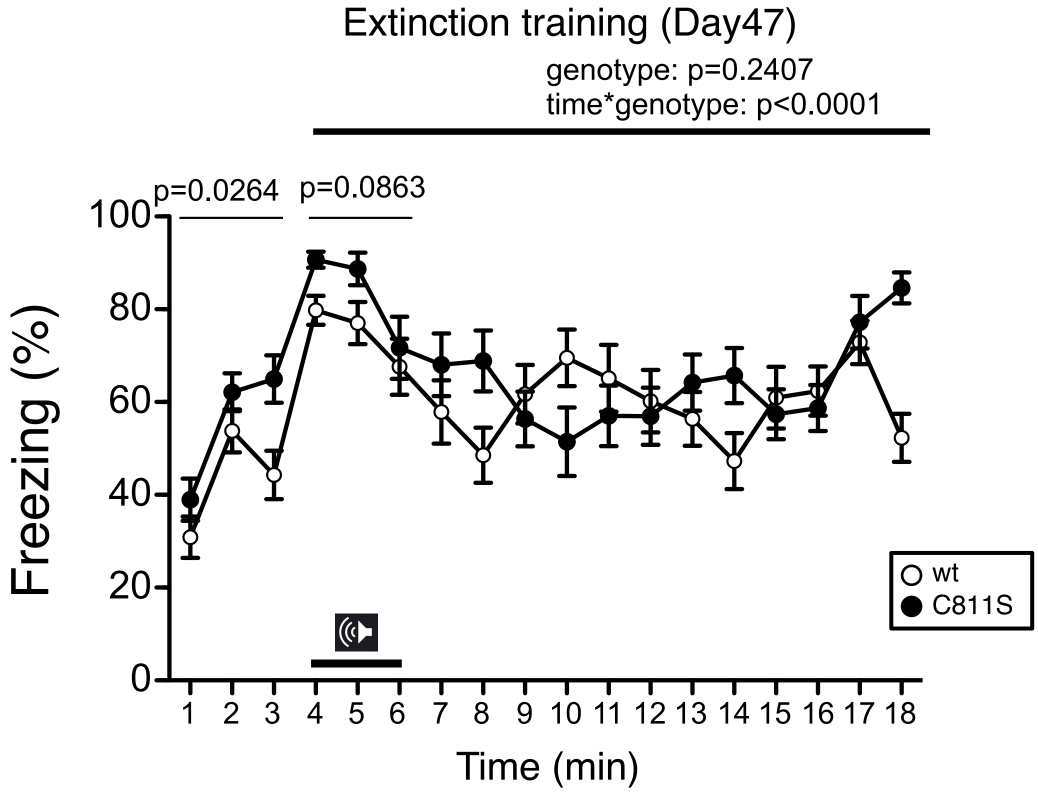

A ABE assay (Amygdala)

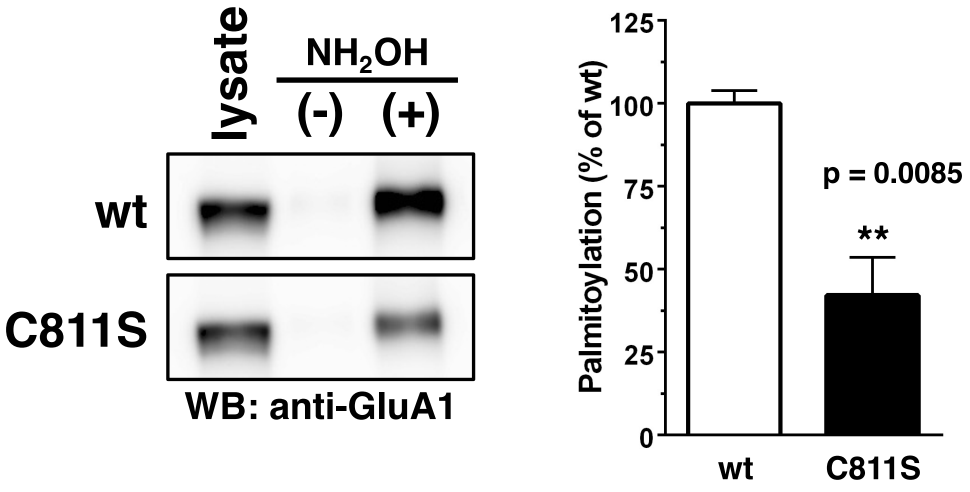

B Protein expression (Amygdala)

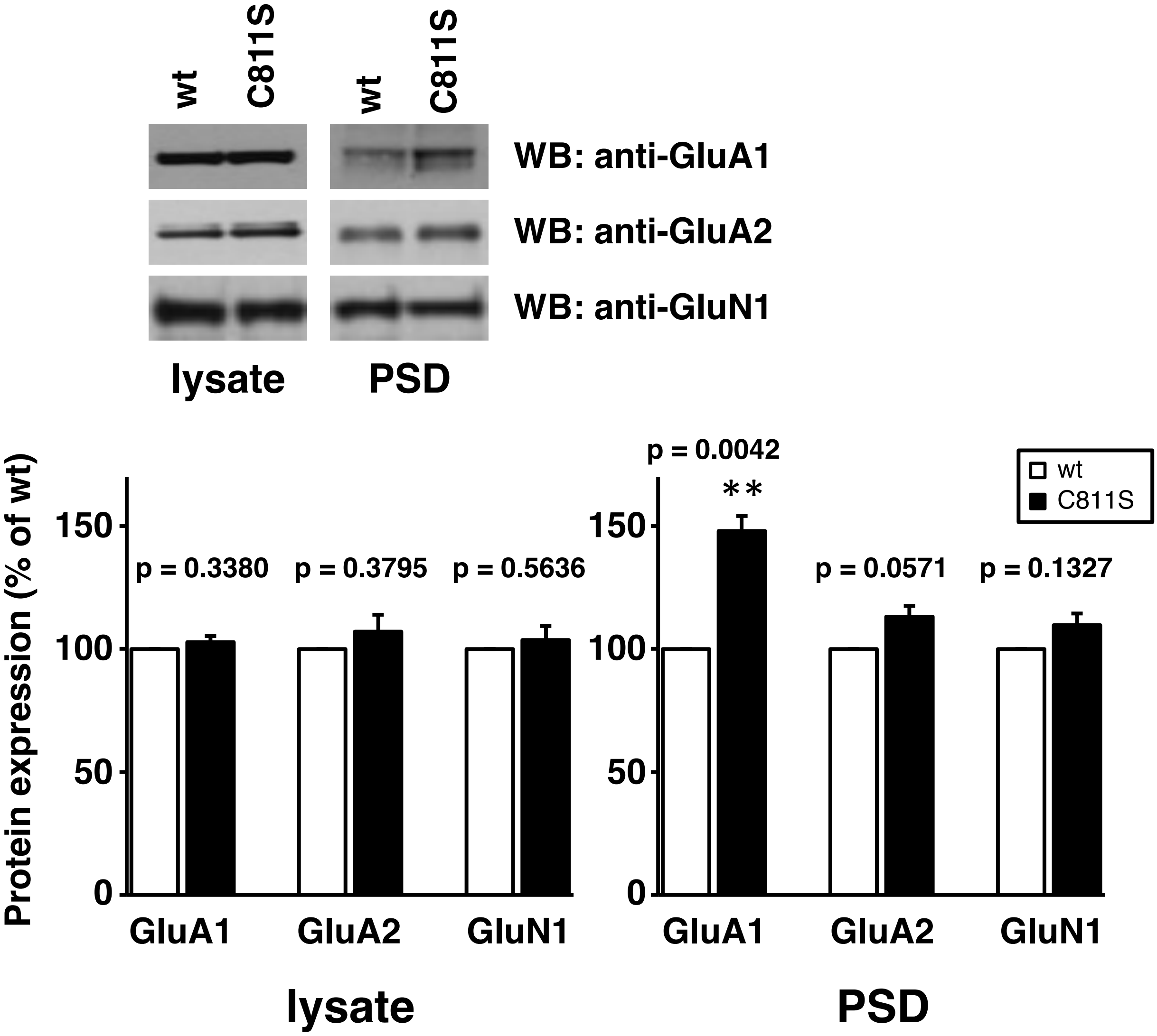

Oota-Ishigaki et al. Supplementary Fig. 7
